# *In vitro* and *in vivo* evaluation of didymin cyclodextrin inclusion complexes: characterization and chemosensitization activity

**DOI:** 10.1080/10717544.2019.1704941

**Published:** 2019-12-20

**Authors:** Qing Yao, Meng-Ting Lin, Qing-Hua Lan, Zhi-Wei Huang, Ya-Wen Zheng, Xue Jiang, Yin-Di Zhu, Longfa Kou, He-Lin Xu, Ying-Zheng Zhao

**Affiliations:** a School of Pharmaceutical Sciences, Wenzhou Medical University, Wenzhou, China;; b Department of Pharmacy, The Second Affiliated Hospital and Yuying Children’s Hospital of Wenzhou Medical University, Wenzhou, China

**Keywords:** Didymin, inclusion complex, multidrug resistance, chemosensitization, 2-hydroxypropyl-β-cyclodextrin

## Abstract

Didymin is a dietary flavonoid that first found in citrus fruits, and possesses antioxidant properties. Our preliminary experiments first discovered that didymin was able to sensitize the resistant cancer cells against chemotherapeutics and combat multidrug resistance. However, its poor aqueous solubility and resultant low bioavailability limit its potentials as an adjuvant phytochemical drug for chemotherapy. Thus, this study prepared the inclusion complex of didymin with β-cyclodextrin and 2-hydroxypropyl-β-cyclodextrin to improve its bioavailability and then evaluate their chemosensitization effects. The didymin inclusion complexes formulation was prepared and their host-guest structure was characterized by FT-IR, PXRD, DSC, and SEM techniques. *In vitro*/*in vivo* results demonstrated that didymin inclusion complex enhanced its water solubility and orally bioavailability. Furthermore, didymin inclusion complex exerted considerable chemosensitivity potency, and improve the anti-tumor effects of chemotherapeutics *in vivo*. Therefore, didymin inclusion complex could provide a safe, effective, economical, and adjuvant drug for future treatment of chemoresistant cancers.

## 1. Introduction

Although many efforts have been made in combating cancer, MDR of chemotherapeutics remains challenging. The extensive application of traditional chemotherapeutics has been limited by intrinsic or acquired resistance to the treatment of breast cancer (Liang et al., 2015). P-gp is a major member in ATP-binding cassette transporter family, which mediated cancer resistance cause the low response of patients toward chemotherapy, and might ultimately leads to treatment failure. Scientists found that compounds could interfere with the P-gp functions and significantly enhance the efficacy of chemotherapy (Khan et al., 2017). For example, verapamil was a calcium channel blocker and recognized as the first-generation P-gp inhibitor since the 1980s. Though verapamil was shown to circumvent MDR successfully, the clinical trials with verapamil as P-gp inhibitors were beset by severe cardiac side effects (Callaghan et al., 2014). Since then, verapamil was removed as a viable option, and nontoxic natural derived P-gp inhibitors that could sensitize the cancer cells is currently considered as one of the most promising alternatives to circumvent MDR. Concurrent use of natural derived P-gp inhibitors and anti-cancer drugs has been widely described to reduce MDR (Kou et al., 2018). Experimental evidence showed that the plant-derived polyphenolic compounds, mainly flavonoids and stilbenes as well as their derivatives, could interfere with the function of multiple chemotherapeutics via P-gp modulation (Yao et al., 2017). The addition of flavonoids with P-gp inhibitory activity could increase the intracellular accumulation of anti-tumor drugs that classified as a P-gp substrate. Also, those dietary flavonoids could be applied to the chemotherapy regime by self-administration before standard chemotherapy.

Didymin, isosakuranetin-7-beta-rutinoside, is one of the dietary glycosides commonly found in mandarin, bergamot, orange, and other fruits or plants. Didymin has long been recognized as a safe, effective, and inexpensive dietary supplement. We have summarized recent advances in pharmacological activities of didymin and related signaling molecules in many diseases (Yao et al., 2018). For example, Huang et al. (2017) provided evidence that didymin has a significant protective effect against CCl_4_-induced hepatotoxicity. In our preliminary experimental exploration, we first discovered that didymin could sensitize the cancer cells and increased anti-tumor ability of co-treated chemotherapeutics. This result suggested that didymin, as a natural fruit flavonoid, could be used as an adjuvant drug for resistant cancer sensitization. However, like many other reported hydrophobic flavonoids, didymin has a low water solubility, which leads to low bioavailability. To explore the feasibility of didymin in cancer treatment, formulation strategies that could address the bioavailability issue is needed. Inclusion complex with cyclodextrins was one important physical strategy to improve the solubility of food and pharmaceutical phytochemicals. The cyclodextrin molecule has a unique amphipathic structure, with a relatively hydrophobic internal cavity and a relatively hydrophilic outer portion, which allows the formation a water-soluble inclusion complex with many hydrophobic drugs to increase the aqueous solubility (Adeoye & bral-Marques, 2017). Therefore, the cyclodextrin inclusion complex strategy can increase the bioavailability and maintain the pharmacological activities of guest molecules, and this has become a practical strategy to formulate the hydrophobic health-promoting flavonoids (Pérez-Abril et al., 2017).

The present study aimed to prepare and characterize dietary didymin inclusion complexes to improve bioavailability and provide an orally adjuvant drug for cancer therapy. The didymin inclusion complexes formulation was prepared via a saturated aqueous solution method, and their host-guest structure was characterized using FT-IR, DSC, PXRD, and SEM methods. More importantly, the chemosensitization effect of didymin and its inclusion complex were evaluated both *in vitro* and *in vivo*, with the mechanisms primarily examined.

## 2. Materials and methods

### 2.1. Materials

HP-β-CD and β-CD were provided by Aladdin Reagent Co., Ltd. (Shanghai, China). Didymin used in the study was obtained by our team (Figure S1, batch number: 20180412, 95.37% of purity). The extraction and isolation procedures were introduced in the Supplementary information. DOX was purchased from Calbiochem (Calbiochem, China). DMSO, MTT and penicillin-streptomycin–neomycin solution were obtained from Sigma (Sigma-Aldrich, St. Louis. MO, USA). RPMI 1640 Medium, DMEM Medium, FBS, and 0.25% trypsin–EDTA were purchased from Gibco (Gibco-BRL, Carlsbad, CA, USA). All other chemicals and buffer solutions were of analytical grade.

### 2.2. Cell lines and cell cultures

HUVEC, H9C2, PC12, MCF-7, and MCF-7/ADR cells are from the Institute of Biochemistry and Cell Biology, Chinese Academy of Sciences. MCF-7, MCF-7/ADR cells were grown in RPMI 1640 medium; other cells were grown in DMEM medium. All the cells supplemented with 10% (v/v) FBS and cultured in a humidified 5% CO_2_ incubator at 37 °C.

### 2.3. Solubility study

Add excess didymin into a different medium (water, ethanol, methanol, 1% β-CD, and 1% HP-β-CD), and keep stirring at room temperature for 72 h. After that, 1.5 mL of the suspension was taken for centrifugation to take the supernatant. After filtering with a 0.45 μM filter membrane (Shanghai Boguang Biotechnology Co., Ltd, Shanghai, China), the concentration of each solution was measured by the HPLC method.

### 2.4. Phase-solubility study

The phase-solubility study was carried out based on the method of Higuchi and Connors. An excess amount of didymin was mixed in an aqueous solution (10 mL) containing specific amounts of cyclodextrins. The mixture was shaken for 72 h at 37 °C in an oven-controlled oscillator. After equilibrium, they were filtered through a 0.45 μm membrane filter. The amount of solubilized didymin was determined using the HPLC method. The apparent stability constant, Ks, of the complex was calculated from the slope of the linear portion as the previously reported (Kaur et al., 2019).

### 2.5. HPLC analysis of the didymin content

An analytical HPLC method was developed to determine the concentration of didymin. The chromatographic conditions are: Agilent HC-C18 column (4.6 mm × 250 mm, 5 μm) (Agilent Technologies, Inc., Santa Clara, CA, USA), the mobile phase was phosphate buffer (pH 6.8)–methanol (45:55), the detection wavelength was 283 nm, the column temperature was 25 °C, and the flow rate was 1.0 mL/min, and. The samples were filtered (0.45 μm) and deaerated before use.

### 2.6. Preparation and characterization of didymin inclusion complex

The didymin inclusion complex was prepared by the saturated aqueous solution method. Briefly, about 0.25 mmoL cyclodextrin (353 mg HP-β-CD or 260 mg β-CD) was weighted and completely dissolved in distilled water (12 mL). After that, around 0.25 mmoL didymin (140 mg) in methanol (12 mL) was added dropwise into the prepared aqueous cyclodextrin solution under stirring in a water bath at 60 °C. In order to ensure that didymin is completely encapsulated in the cavity of the HP-β-CD or β-CD, the solution was equilibrated for 6 h under constant temperature (60 °C) magnetic stirring. After that, the methanol solvents were evaporated under reduced pressure, and the acquired inclusion complex liquid was frozen at −20 °C overnight. Finally, the frozen inclusion complex was lyophilized until a constant mass. For comparison, the physical mixture of didymin and cyclodextrin, including didymin-β-CD and didymin + β-CD, were also prepared by mixing in a solid state in the mortar.

### 2.7. Characterization of the didymin inclusion complex

#### 2.7.1. FT-IR spectroscopy

The infrared (IR) spectrum of didymin, cyclodextrin, the didymin inclusion complex, and their physical mixture was obtained according to the KBr disc method, using an FT-IR spectrometer (Shimadzu, Japan). The scans were executed with a resolution of 4 cm^−1^ and from 4000 cm^−1^ to 400 cm^−1^.

#### 2.7.2. PXRD study

The solid-state properties of didymin, cyclodextrin, the didymin inclusion complex, and their physical mixture were studied by PXRD using a PW1729 Philips vertical scanning diffractometer (Philips Electronic Instruments, New York, NY, USA). These samples were irradiated using monochromatized Cu-Kα radiation operating at 40 kV and 450 mA and analyzed in the angle range of 3–40 2*θ* at a step of 0.02°/min.

#### 2.7.3. DSC study

DSC curves of didymin, cyclodextrin, the didymin inclusion complex, and their physical mixture were studied by a Mettler TA 3000 DSC analyzer (Greifensee, Switzerland). The samples were placed in a sealed aluminum sample pan, and the temperature was increased from 40 °C to 240 °C with a constant rate of 10 °C/min. An empty sealed pan was used as a reference.

#### 2.7.4. Scanning electron microscope (SEM)

The morphological feature of didymin, cyclodextrin, the didymin inclusion complex, and their physical mixture was examined in the SEM (Inspect S50, Japan). The pictures were recorded at an excitation voltage of 25 kV, a magnitude of 3000–12,000× and focusing from 10 to 14.1 mm.

#### 2.7.5. Magnetic resonance imaging (NMR)

The NMR spectra of didymin, cyclodextrin, the didymin inclusion complex, and their physical mixture were taken on a JEOL JNM-ECS-400 (JEOL, Tokyo, Japan). Except didymin were dissolved in CDCl_3_, other samples of 8 mg were dissolved in 0.6 mL D_2_O.

### 2.8. Pharmacokinetic study

All animal experiments were carried out under the Guide for Care and Use of Laboratory Animals and approved by the approval of the Ethics Committee of Wenzhou Medical University. Male SD rats (3–5 weeks old, Shanghai Laboratory Animal Center, China) weighing 220–250 g (*n* = 6 per group) were studied in the pharmacokinetic study. Free didymin and two types of didymin inclusion complexes (25 mg/kg, respectively) were orally administered with sterilized water. Then blood samples were collected from the orbit venous plexus at predetermined time intervals. The blood samples were then centrifuged at 15000 r/min for 10 min, then dried at 40 °C under nitrogen flow to solid-phase extraction. Methanol was added to dissolve the residue. Then the concentration of didymin in plasma was determined by HPLC methods and calculated the pharmacokinetic parameters.

### 2.9. *In vitro* chemosensitivity evaluation

#### 2.9.1. Cytotoxicity assay

The cytotoxicity of didymin on various cell lines was examined by MTT assay as previously reported (Yao et al., 2019). The didymin solution was prepared by dissolving in DMSO at different concentrations and diluted with the fresh medium right before the treatment. The didymin inclusion complex was dispersed in the sterilized water and then diluted with the fresh medium before treatment. Experiments involving healthy cells (HUVEC, H9C2, and PC12) by culturing 100 μL cell suspension (5 × 10^4^ cell/mL) in 96 well plates. After 24 h incubation, the didymin or didymin inclusion complexes contained medium was used to replace the overnight medium and cultured for 48 h, respectively. After that, the MTT solution at the concentration of 5 mg/mL was added to each well. After 4 h incubation, the medium was removed, and the MTT formazan was dissolved by DMSO. Then the DMSO solution is measured using a microplate reader with the absorbance wavelength at 570 nm. The untreated cells were used as control. The cell viability (%) was calculated as follows, cell viability (%) = A_sample_/A_control_ ×100.

The MCF-7 and MCF-7/ADR cells cytotoxicity of the combination of didymin and doxorubicin were evaluated by MTT assay, as mentioned above.

#### 2.9.2. Intracellular uptake study

The effect of didymin on the intracellular uptake of the P-gp substrate was conducted as previously reported (Dai et al., 2016; Yao et al., 2019). Briefly, MCF-7/ADR cells were seeded in a 6-well plate at 4 × 10^5^ cell/mL overnight before the experiment. Then the cancer cells were incubated with DOX (10 μM) at a concentration with or without didymin formulations for 4 h. Then, the treated cells were washed with ice-cold PBS three times and recorded under fluorescence microscopy (Nikon, Tokyo, Japan). In an independent study, after treatment, the cells were washed with ice-cold PBS and then lysed. Then acetonitrile was added into the cell lysate to extract the didymin via protein precipitation (PP)/extraction method. The didymin concentration in the supernatant was determined by the previously validated HPLC method.

#### 2.9.3. Calcein-AM extrusion assay

The effect of didymin and its inclusion complex on the efflux function of P-gp transporter was studied using calcein-AM extrusion assay as reported (Dai et al., 2016). Briefly, MCF-7/ADR cells were seeded in a 6-well plate at 2 × 10^5^ cell/mL overnight before the experiment. The cancer cell was incubated with verapamil or didymin formulations for 30 min and then treated with calcein-AM for another 30 min. Then, the treated cells were washed with ice-cold PBS three times and recorded under fluorescence microscopy. In an independent study, after treatment, the cells were washed, trypsinized, and collected by centrifuge. The collected cells were then washed with PBS and analyzed by flow cytometry (BD Bioscience, Franklin Lakes, NJ, USA). The data was collected with 1 × 10^5^ events counter per samples

### 2.10. Hemolysis evaluation

A 2% red blood cell suspension was used to evaluate the hemolytic activity of the didymin inclusion complex. Didymin/HP-β-CD and didymin/β-CD (10 µg/mL, 100 µg/mL, 1000 µg/mL) were used for experiments. Distilled water and saline solution were used as the positive and negative control, respectively. The mixture was incubated at 37 °C for 4 h, and the absorbance of the supernatant was measured at 570 nm.

### 2.11. Acute toxicity study

An acute toxicity test was conducted as previously reported (Kandhare et al., 2016). In brief, the BALB/c mice weighing about 18–22 g were administrated of didymin at a single dose of 250 mg/kg using oral gastric gavages. The experimental mice were observed for 14 days after oral administration to observe any toxicity sign. On 15th day animals were sacrificed, and the major organs were collected, fixed, and sliced for H&E staining.

### 2.12. *In vivo* antitumor efficacy

To further investigate the chemosensitivity of didymin inclusion complex, female BALB/c nude mice (5–6 weeks old, Shanghai Laboratory Animal Center, China) were injected subcutaneously with MCF-7/ADR cells (10^7^ cells/per mouse). When the tumor volume reached 50–100 mm^3^, the mice were randomly divided into three groups (*n* = 5) as follows: (1) Control group, 0.9% saline (0.2 mL/kg/day, i.p.); (2) DOX group, DOX (5 mg/kg/3 days, i.p.); (3) DOX + Didymin/HP-β-CD group, DOX (5 mg/kg/3 days, i.p.) and Didymin/HP-β-CD (20 mg/kg/day, i.g.). Didymin/HP-β-CD was given by oral gavage (i.g.) while DOX was intraperitoneal injected (i.p.). DOX injection was performed 30 min after the didymin was given by gavage. The mouse weight and tumor volume were measured every day. The tumor volume was obtained using the formula (Yao et al., 2019). On day 14, the mice were sacrificed, and tumor tissues were collected, fixed and sliced for pathological examination (H&E staining, Ki67 immunohistochemical staining, and TUNEL assay).

### 2.13. Statistical analysis

The difference between the groups was analyzed using the student’s *t*-test. The values of *p* < .05 was considered statistically significant.

## 3. Results and discussion

### 3.1. Solubility and phase-solubility studies

The solubility of didymin was studied in water, methanol and 1% cyclodextrin solution. The lowest solubility value was recorded in water as 0.107 ± 0.12 mg/mL, while it is 11.21 ± 1.19 mg/mL in methanol. It is consistent with the previously reported study that didymin has low solubility in aqueous solution (Hung et al., 2010). As expected, adding cyclodextrin into the water dramatically increased the didymin solubility. In the 1% HP-β-CD solution, the solubility of didymin was increased to 0.735 ± 0.37 mg/mL, while the solubility in ethanol only reached 1.242 ± 0.72 mg/mL. With the presence of HP-β-CD and β-CD, the aqueous solubility of didymin was significantly increased 6.9 times and 4.7 times compared to that of free didymin. It was indicated that the cyclodextrin, like β-CD and HP-β-CD, could solubilize the didymin, which is closely related to the formation of the inclusion complex between the didymin and cyclodextrin.

The phase-solubility study of didymin and cyclodextrin were investigated (Figure S2). The results indicated that aqueous solubility of didymin was linearly influenced by the presence of cyclodextrin, which reflected in figure S2 that the solubility dramatically increased as the cyclodextrin concentration went up. The calculated Ks values for didymin/HP-β-CD and didymin/β-CD inclusion complexes were 583.3 M^−1^ and 295.8 M^−1^, respectively, indicating that the inclusion tendency of guest didymin with host cyclodextrin was appropriate (Tačić et al., 2014). The higher value of Ks was observed in the HP-β-CD group, demonstrating that the hydrophilization of β-CD enhance the complexation efficiency of the inclusion complex (Lima et al., 2019).

### 3.2. Characterization of didymin inclusion complexes

**Figure 1.**
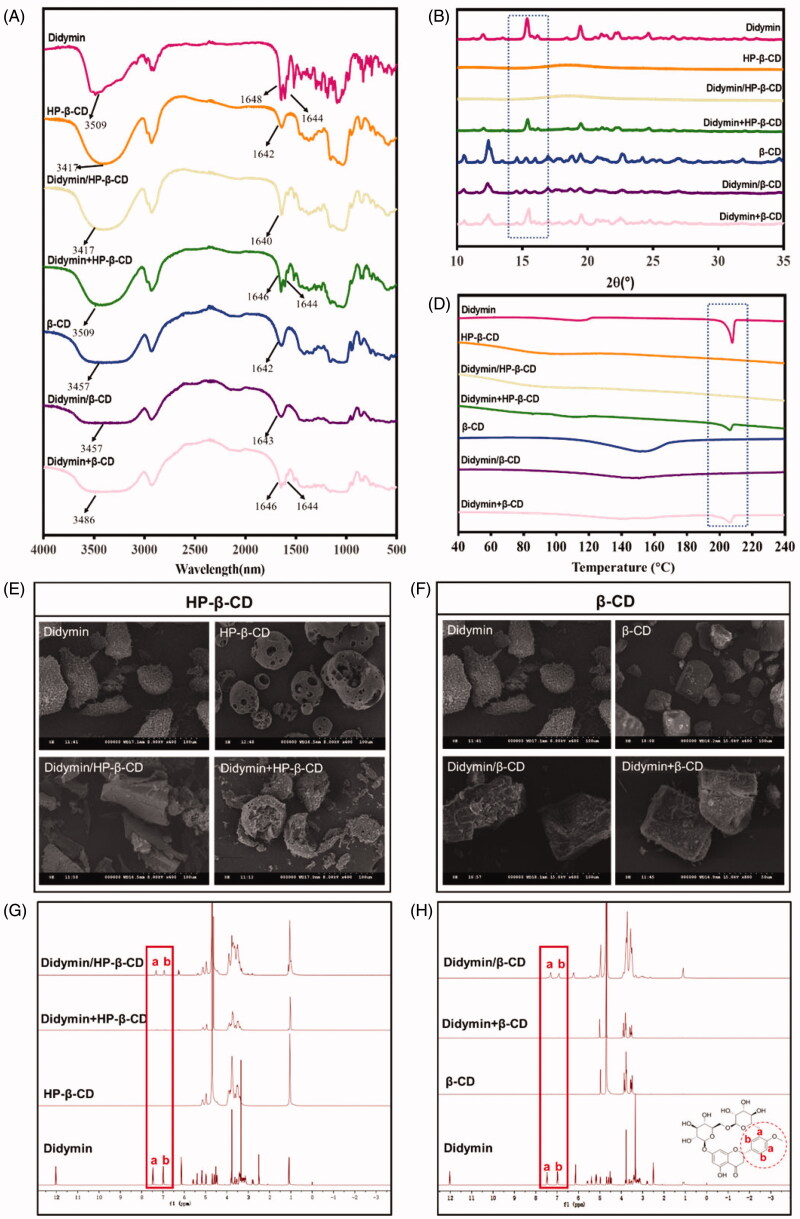
Characterization of didymin inclusion complex. FT-IR spectra (A), PXRD diffractogram (B), DSC thermograms(C), SEM micrographs (D, E) and NMR spectrum (F, G) of the of didymin, cyclodextrin (HP-β-CD and β-CD), didymin inclusion complex (didymin/HP-β-CD and didymin/β-CD) and physical mixture (didymin + HP-β-CD and didymin + β-CD).

The didymin cyclodextrin inclusion complex was prepared using a classic saturated aqueous solution method with two types of cyclodextrin, HP-β-CD, and β-CD. To verify the formation of didymin inclusion complex and the host-guest structure, we adopted multiple characterization methods, including FT-IR spectrum (Figure 1(A)), PXRD spectrum (Figure 1(B)), and DSC spectrum (Figure 1(C)).

The FT-IR technique could identify the changes of characteristic peak position, shape and frequency, thereby used to study the host-guest interaction in the inclusion complex (Pu et al., 2018). As presented in Figure 1(A), the FT-IR spectrum of pure didymin showed characteristic stretching frequency of C=C bond, C-O bond with the appearance of a double peak at 1644 cm^−1^ and 1648 cm^−1^. Also, there was a broad and robust peak at 3509 cm^−1^, which represents -OH valence vibration in the didymin structure. As described in other literature (Lima et al., 2019), a strong and broad peak at 3417 cm^−1^ of -OH stretching vibration and a single peak at 1642 cm^−1^ were observed in the FT-IR spectrum of cyclodextrin. The spectrum of the didymin inclusion complex appeared to be very similar to those of cyclodextrin, which exhibits many polar groups (−OH, C=O), resulting in a broad absorption band. At the same time, some specific peaks of didymin were absent or shifted; only one single peak appeared around 1642 cm^−1^. However, the FT-IR spectrum of the physical mixture was solely presented as a simple superposition of the didymin and cyclodextrin. The significant difference in the characteristic peaks of didymin presented in the FT-IR spectrum of didymin and didymin inclusion complex confirmed that the guest compounds had entirely or partially entered the cavity of cyclodextrin and formed the inclusion complex.

The formation of the inclusion complex between the cyclodextrin and crystalline guest molecule could be confirmed with the absence of a crystalline state of guest molecules after complexation with cyclodextrin. Therefore, PXRD study was commonly used to identify the physical state of guest molecules and characterize the inclusion complex (Figure 1(B)). Didymin exhibited sharp and strong diffraction peaks, indicating that didymin existed as a crystalline state in the solid state. Meanwhile, the diffraction spectrum of HP-β-CD only presented two broad peaks, indicating an amorphous state (Michalska et al., 2017). The diffraction pattern of the physical mixture (didymin + HP-β-CD) was only a superposition of the patterns of didymin and HP-β-CD, which confirmed that the physical state of didymin was unchanged in the physical mixture. However, the characteristic sharp peaks of didymin disappeared in the patterns of didymin/HP-β-CD inclusion complex, indicating that didymin was embedded in the cavity of HP-β-CD by the hydrophobic effect and lost its crystalline state. Similarly, the patterns of the physical mixture (didymin + β-CD) was essentially a simple superposition of patterns of pure didymin and β-CD. Moreover, the diffraction pattern obtained from the didymin/β-CD inclusion complex was identical to that of β-CD. These results indicated that the physical states of didymin before and after inclusion complex formulation were different and confirmed the formation of the didymin inclusion complex. We also conducted the PXRD study of didymin inclusion complex after three months storage (Figure S4). The result showed that the diffraction spectrum of didymin inclusion complex remained unchanged after storage, indicating that the didymin and cyclodextrin could form a stable inclusion complex.

It is consistent with the FT-IR, and PXRD data, the DSC curves (Figure 1(C)) of the inclusion complex were very different from that of the didymin and cyclodextrin, as well as the physical mixture. Didymin exhibited a high-intensity peak at 207 °C in the DSC curve, further confirming the crystal state of didymin in Figure 1(B). The amorphous state of the cyclodextrin was also shown in the DSC curves of both HP-β-CD and β-CD with a shallow, grooved flat line appeared at 102 °C. As expected, the DSC curves of the physical mixture were identical to a superposition of DSC curves of didymin and cyclodextrin. However, the endothermic peak at 207 °C of didymin was disappeared in the DSC curves of the inclusion complex, indicating the didymin presented in the relatively hydrophobic cavity, thereby forming an amorphous complex. The difference in the DSC curves of the didymin and its inclusion complex could be attributed to the interaction between the guest molecule didymin and host molecule cyclodextrin, which is accordance with the FT-IR result (Figure 1(A)). The DSC results further confirmed that the didymin molecules entered the cyclodextrin cavity and formed an inclusion complex.

SEM is another widely used technique to characterize the inclusion complex by visualizing the surface properties of the drugs and formulations (Qiu et al., 2014), and also performed here (Figure 1(D,E)). the SEM micrograph of didymin was irregular particles and presented as needlelike crystals of different sizes. Similar to the previous literature (Huang et al., 2016), HP-β-CD had a typical hollow, solid, and amorphous structure (Figure 1(D)). As expected, the small needlelike crystals of didymin and the amorphous microparticles of HP-β-CD were coexisted in the SEM images of their physical mixture, indicating that they were merely mixed. Didymin adhered to the surface of the cyclodextrin when physically mixing didymin and cyclodextrin powder, and they did not form an association. However, for the didymin/HP-β-CD inclusion complex, the needlelike crystal of didymin disappeared as the cavity of the cyclodextrin was filled in the SEM images, exhibiting the block-like structural features with irregular sizes. The dramatic morphological change attribute to the host-guest interaction between didymin and HP-β-CD. Similar results were also found in the didymin/β-CD inclusion complex (Figure 1(E)). Due to the formation of didymin/β-CD, the didymin did not exist as crystal in the inclusion complex anymore. The SEM micrograph further confirmed the PXRD (Figure 1(B)) and DSC (Figure 1(C)) results.

NMR is a technique that provides evidence for the inclusion of a guest molecule into the cyclodextrin cavity (Teixeira et al., 2014). Insoluble particles will not show up in a solution NMR spectrum. Didymin has poor aqueous solubility in water, thus the NMR spectrum of didymin was detected in CDCl_3_ solvent. All other samples were detected in D_2_O solvent. The didymin inclusion complex (didymin/HP-β-CD and didymin/β-CD) showed the characteristic peaks of didymin at δ (ppm) ∼7.0 and δ (ppm) ∼7.5 in D_2_O solvent, which is consistent with the chemical shift in the NMR spectrum of didymin in the CDCl_3_ solvent. Instead, the signals around the δ (ppm) = 7.0 ∼ 7.5 in physical mixture samples are very weak. Therefore, didymin in the inclusion complex samples can be detected in the NMR spectrum, which also confirms the successful preparation of the inclusion complex.

### 3.3. *In vivo* pharmacokinetic studies

**Figure 2.**
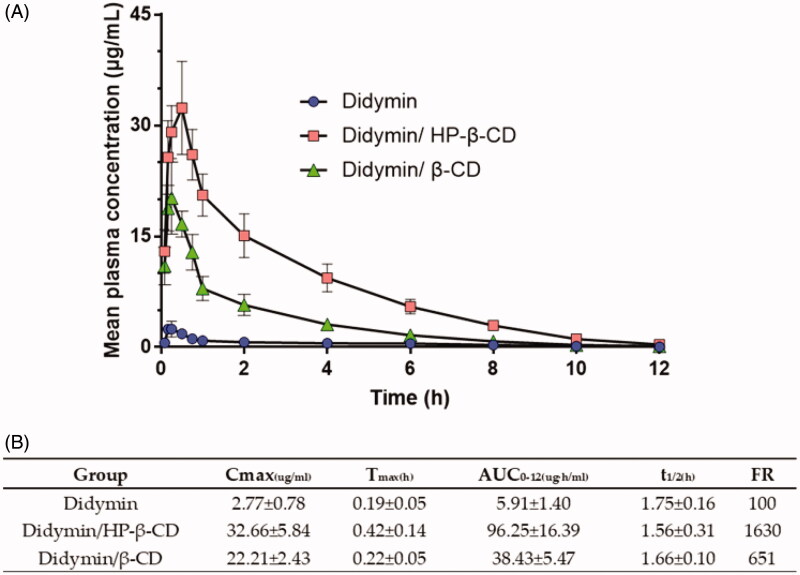
*In vivo* pharmacokinetic study of oral administration of didymin inclusion complex in SD rats. (A) Mean plasma concentration-time profiles. Each value represents the mean ± SD (*n* = 6). (B) *In vivo* pharmacokinetic parameters.

Then, we investigated the pharmacokinetic profiles of oral didymin inclusion complex, a new preparation of didymin, in rats. Figure 2(A) presented the plasma concentration versus time curve following the oral administration of the didymin and didymin inclusion complex (25 mg/kg). The pharmacokinetic (PK) parameters are summarized in Figure 2(B). The extent of absorption, as indicated by AUC_0–12 h_, was 5.91, 96.25, and 38.43 ug·h/mL, respectively, for the didymin, didymin/HP-β-CD, and didymin/β-CD. It indicated that the formulation strategy using the inclusion complex could significantly increase the *in vivo* absorption of didymin. However, the bioavailability of inclusion complex prepared by didymin and HP-β-CD was nearly 3 times higher than that of inclusion complex prepared by didymin and β-CD. The *in vivo* bioavailability data echo the *in vitro* solubility results. The Cmax of the didymin, didymin/HP-β-CD, and didymin/β-CD were 2.77, 32.66, and 22.21 ug/mL, respectively. These data further suggested that the drug had good gastrointestinal permeability and that the absorption of the drug molecules was rapid if they were solubilized. The difference of bioavailability between didymin/HP-β-CD and didymin/β-CD could be attributed to the difference in the potent solubility of two types of cyclodextrin. The higher solubility of HP-β-CD leads to a faster dissolution of didymin/HP-β-CD and thereby increasing the extent of *in vivo* absorption (Garrido et al., 2018). It was also noticeable that the Tmax for all three formulations were all less than 0.5 h, which also provided the information for the future preclinical studies of combination use of didymin and chemotherapeutics in cancer treatment. The time interval between the didymin administration and the cancer treatment might need to be adjusted based on the pharmacokinetic profiles of both didymin and chemotherapeutics.

### 3.4. Safety assessment of didymin inclusion complexes

**Figure 3.**
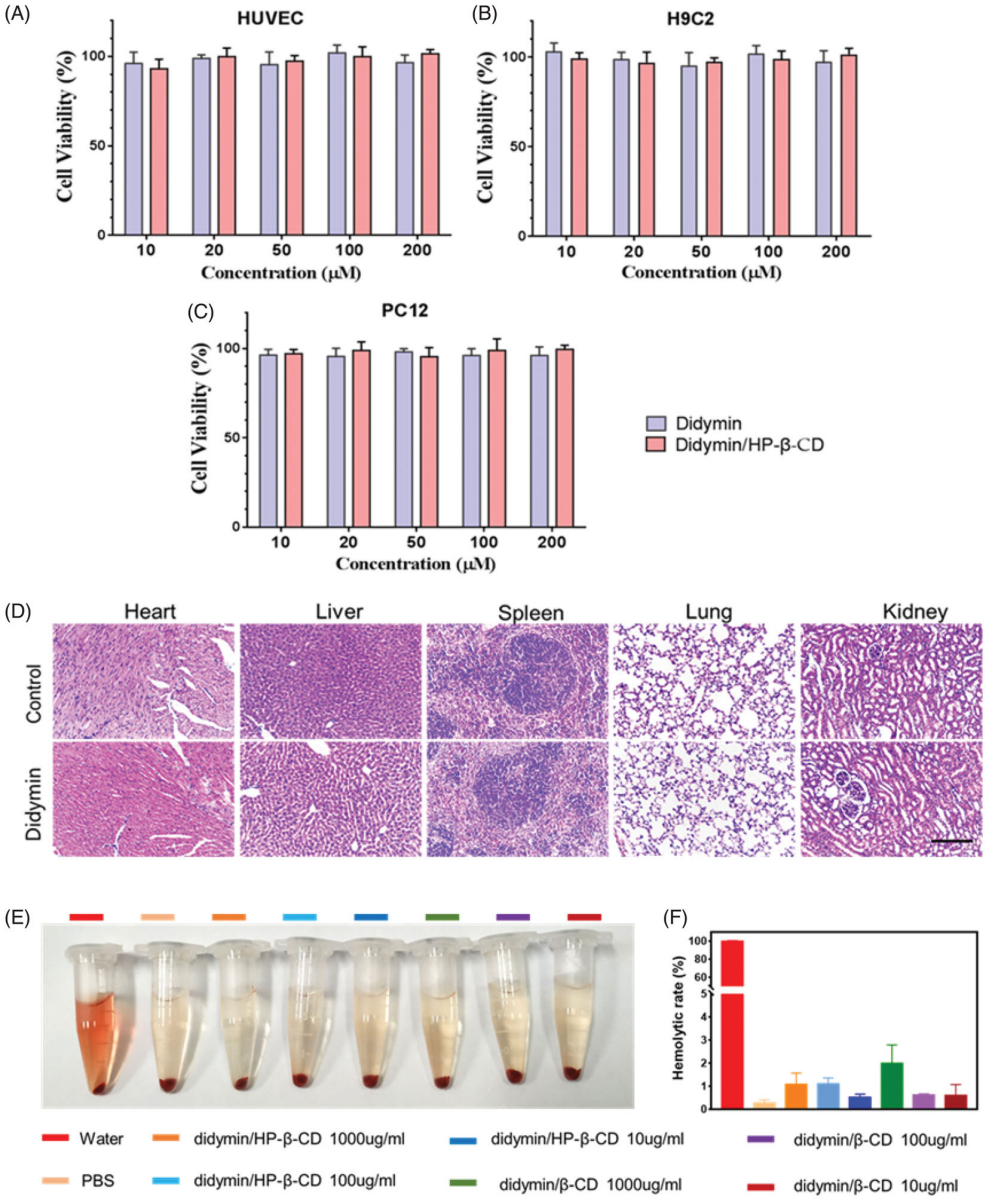
Toxicity of didymin and didymin inclusion complex on health cell lines: HUVEC cells (A), H9C2 cells (B), and PC12 cells (C). (D) Acute toxicity test, oral administration of Didymin (250 mg/kg) and normal saline, H&E staining assay of major organs after 14 days. Scale bar: 100 µm. Hemolysis evaluation of didymin inclusion complexes by UV-Vis spectrophotometry *in vitro*. Photograph (E) and hemolytic rate (F) of red blood cells treated with different groups. Data are presented as mean ± SD (*n* = 3).

The cellular toxicity of didymin has been previously studied in several reports (Hung et al., 2010; Singhal et al., 2012). To further study its feasibility as an oral adjutant drug for cancer therapy, multiple healthy cell lines were chosen in this study, including HUVEC, H9C2, and PC12 cells. The cellular toxicity of didymin, as well as didymin inclusion complex, was determined by MTT (Figure 3(A–C)). After the incubation with didymin contained medium for 48 hours, the cell viability remained higher than 90% at all tested concentrations (10 μM∼200 μM). The hemolysis rate of the inclusion complex was also evaluated *in vitro* (Figure 3(E,F)). The results showed that there was severe hemolysis in the pure water of the positive group. No significant hemolysis was seen in the didymin inclusion complex, which indicated the high biosafety of the didymin inclusion complexes formulation (Figure 3(E,F)). Additionally, in the acute toxicity study, we observed that orally administrated didymin and its inclusion complexes at high dosage (250 mg/kg) did not show any abnormal behaviors and weights change during the observation period. The H&E staining of major organs (Figure 3(D)) also showed no signs of any toxicity, demonstrated the high safety of dietary didymin in the tested dose. This result indicated that didymin is highly safe, which also paved the way to become an adjuvant drug for cancer treatment without causing safety issues.

### 3.5. Chemosensitization study of didymin inclusion complexes *in vitro*

**Figure 4.**
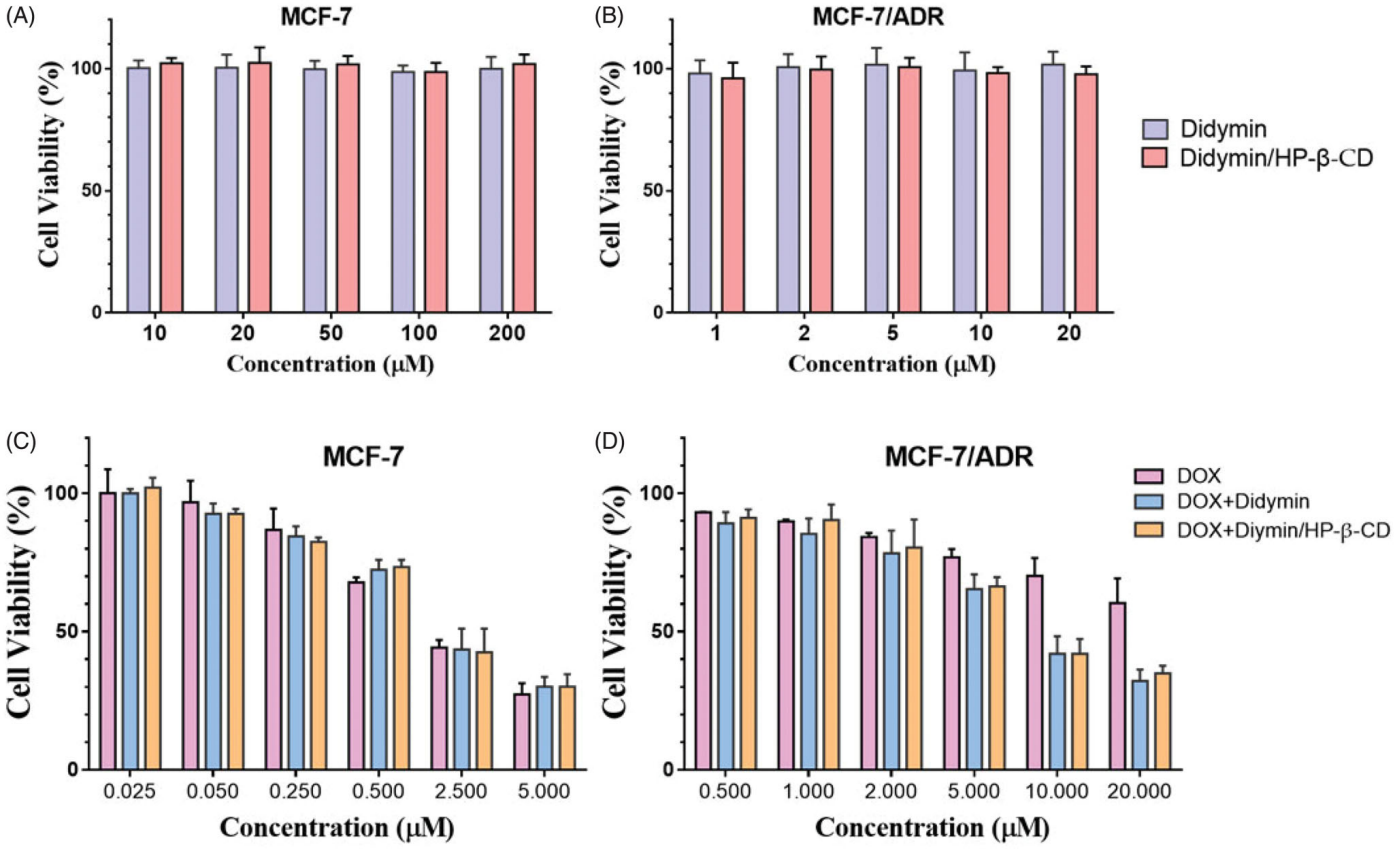
The cytotoxicity (A, B) and chemosensitivity (C, D) of didymin inclusion complexes on the tumor sensitive cells (A, C) and resistant cells (B, D).

The chemosensitivity of didymin and its inclusion complex was evaluated on the drug-resistant MCF-7/ADR cells, and drug-sensitive MCF-7 cells used as a control. In our preliminary study, we discovered that didymin could dramatically increase the cytotoxicity of DOX on the resistant cancer cells. With the high safety of didymin (Figure 3), it is of great potentials to develop an adjuvant formulation for cancer therapy without any side effects. The inclusion complex strategy increased the solubility and bioavailability of didymin, but whether it will affect its pharmacological effect on the cancer cells is unknown. We first evaluated the cytotoxicity of didymin inclusion complexes on sensitive tumor cells (Figure 4(A)) and resistant tumor cells (Figure 4(B)). It is consistent with the above toxicity results that didymin or didymin inclusion complex alone did not exert toxicity on cancer cells in our tested concentrations. Then we study the chemosensitivity of didymin on the sensitive cells (Figure 4(C)) and resistant cells (Figure 4(D)), where DOX was used as a model chemotherapeutic drug. It is predictable that DOX showed dose-dependent cytotoxicity on both two cell lines, while the cellular response is different. MCF-7 cells were more sensitive to drug exposure, and the IC 50 was 2.26 ± 0.47 μM. On the contrary, the IC50 value of free DOX against drug-resistant MCF-7/ADR cells was even higher than 20 μM. The difference in cellular response confirmed the resistance of MCF-7/ADR cells to chemotherapeutics. While didymin alone did not exert apparent cytotoxicity on cells, it sensitizes the resistance cells in the exposure to chemotherapeutics. As shown in Figure 4(D), the combination of DOX and didymin achieved a significantly lower IC50 value at 6.50 ± 0.84 μM. The formation of didymin inclusion complexes did not alter the chemosensitivity of didymin, which also significantly increased the cytotoxic activity of DOX on the MCF-7/ADR cells, with IC50 at 5.67 ± 0.69 μM. It was indicated that adding didymin into chemotherapeutic regimen could significantly increase the anti-tumor efficacy.

**Figure 5.**
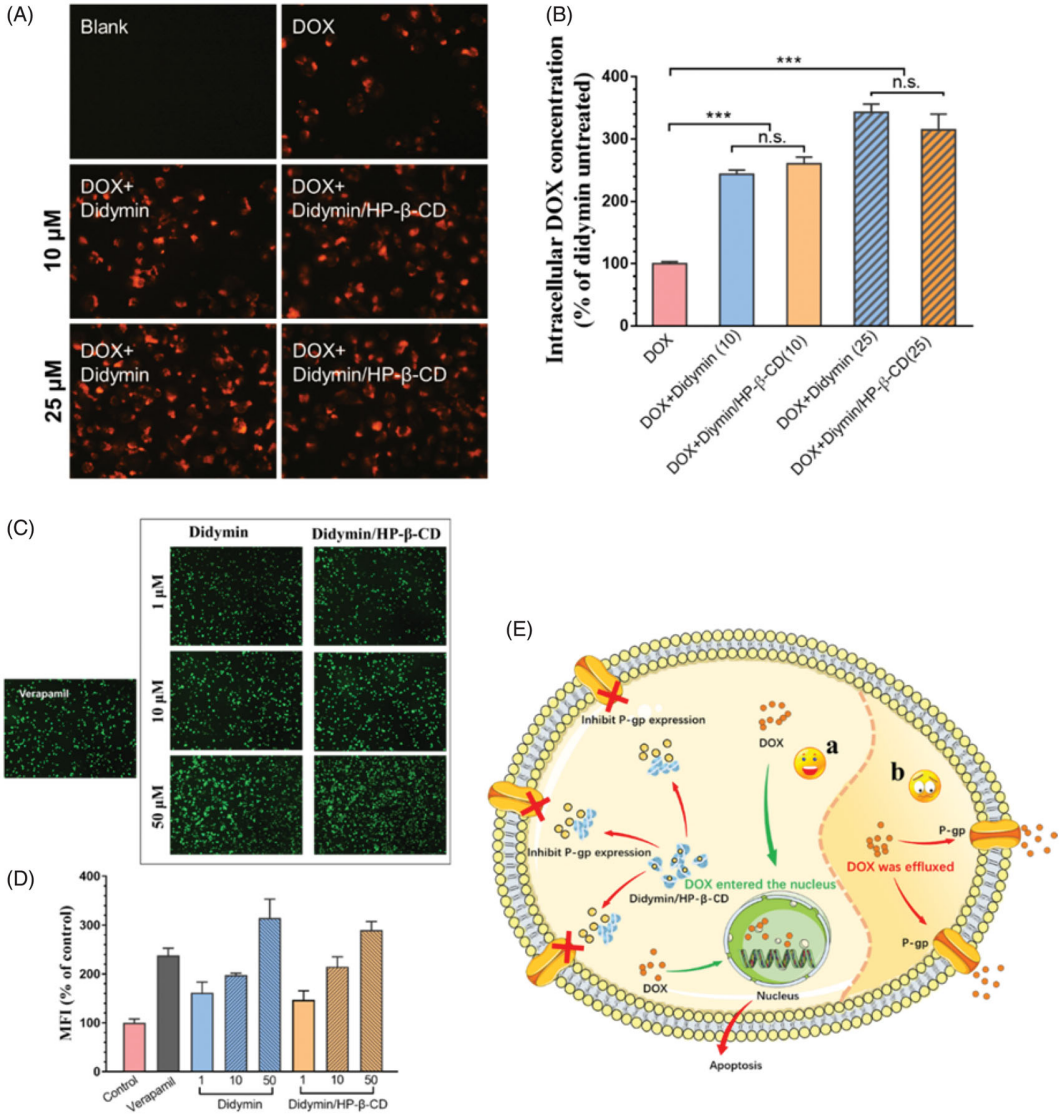
Fluorescence microscopy images (A) and flow cytometers (B) of the cellular uptake of DOX with or without didymin on resistant tumor cells. Original magnification: 20×. ** represents *p* < .01, *** represents *p* < .001. Fluorescence microscopy images (C) and flow cytometers (D) of resistant tumor cells incubated with Calcein-AM with or without additives for 1 h. Original magnification: 4×. (E) Schematic diagram of chemosensitization mechanisms of Didymin/HP-β-CD. In the presence of Didymin/HP-β-CD, DOX could increase the intracellular accumulation and enter the nucleus to induce apoptosis (C-a), but in the opposite case, most of DOX was effluxed out of the cell through P-gp transporter (C-b).

To further understand the chemosensitivity of didymin and its inclusion complex, we evaluated the cellular uptake of DOX with or without didymin on MCF-7/ADR cells by fluorescence microscopic technique (Figure 5(A)) and quantitative analysis (Figure 5(B)). DOX is a well-reported P-gp substrate, which could be easily effluxed by the resistant cells, causing low cellular bioavailability. Therefore, it is reasonable to observe a relative dark red fluorescence intensity of DOX. However, in the presence of didymin, either free drug or inclusion complex form, the fluorescence intensity was significantly stronger (Figure 5(A)). With the concentration of didymin increased, the fluorescence intensity increased as well, indicating a concentration-dependent uptake enhancement. The quantitative results of intracellular DOX content in Figure 5(B) were consistent with the fluorography results. Therefore, the DOX cellular uptake results showed that didymin could increase the DOX intracellular accumulation, which explained the dramatically increased cytotoxicity of DOX with the presence of didymin (Figure 4(D)).

With the significantly increased DOX uptake on resistant cells, we hypothesized that the P-gp inhibitory effect might be one of the chemosensitivity mechanism of didymin. We studied the effect of didymin and its inclusion complex on Calcein-AM uptake (Figure 5(C,D)). Calcein-AM is a well-known hydrophobic P-gp substrate, which could be converted to hydrophilic fluorescent form (calcein) via the intracellular esterase. Thus, the intracellular calcein fluorescence could reflect the P-gp efflux capability and commonly used to assess the P-gp inhibitory effects of various compounds. For comparison, a classic P-gp inhibitor, verapamil, was used as a positive control. With the co-treatment of didymin or didymin inclusion complex, the intracellular calcein fluorescence exhibited a dose-dependent increase as shown in Figure 5(C,D), which indicated that didymin or didymin/HP-β-CD could significantly inhibit the function of P-gp transporter. This effect was consistent with the intracellular DOX content in Figure 5(A,B). The Calcein-AM assay result demonstrated that the chemosensitivity of didymin was closely related to P-gp inhibition (Figure 5(E)). More importantly, the formation of didymin inclusion would not affect the cancer sensitization effect of the potent drug.

### 3.6. Chemosensitization study of didymin inclusion complexes *in vivo*

**Figure 6.**
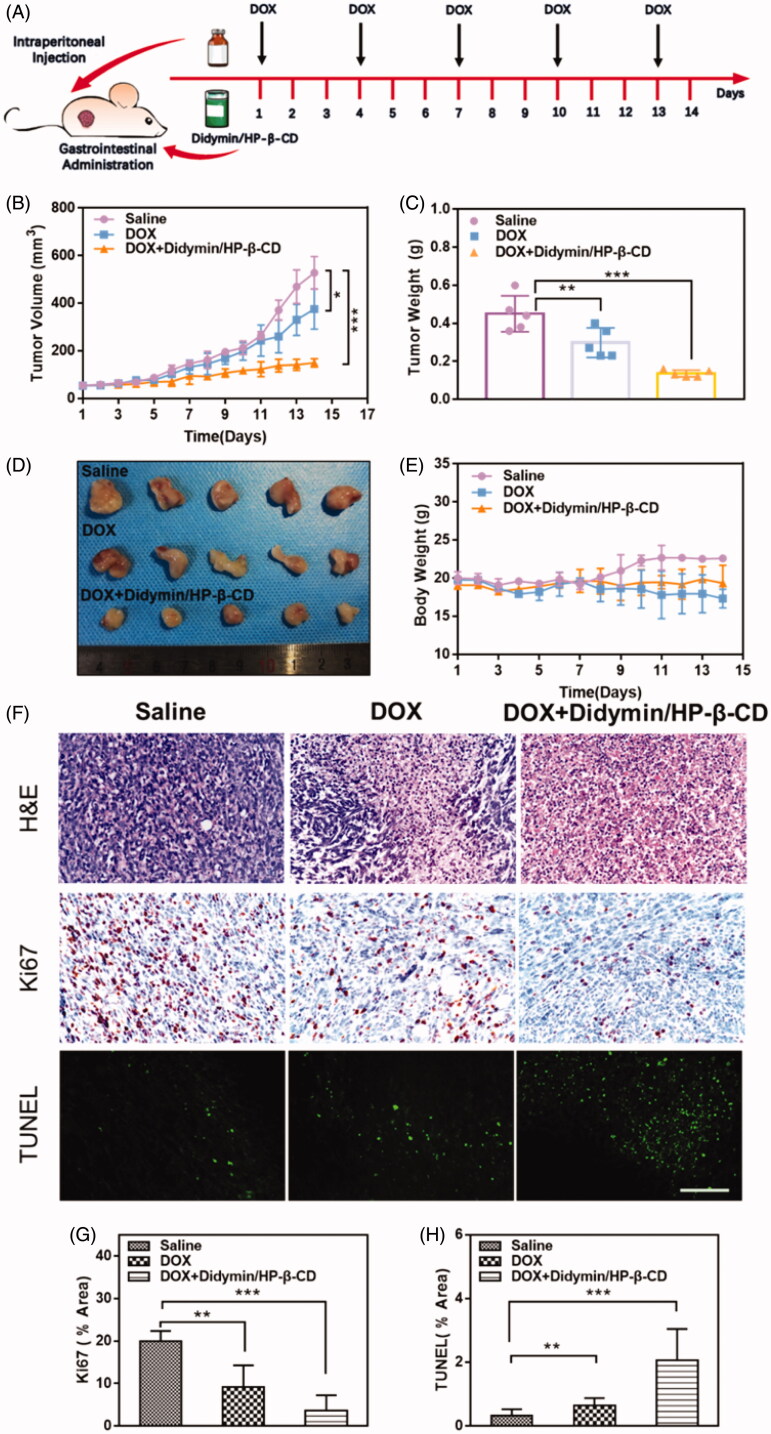
*In vivo* anti-tumor efficiency of chemotherapeutic plus Didymin/HP-β-CD therapy in nude mice bearing MCF/ADR tumors. (A) The schematic diagram for *in vivo* chemosensitization study of Didymin/HP-β-CD for chemotherapy. (B) Tumor volume change curves. The tumor weight and images of the excised tumors on day 14. (E) Bodyweight change mice during treatment. (F) Histological analysis of the tumor tissues receiving different cancer therapies on day 14. Ki67- (G) and TUNEL-(H) positive level were measured from Immunohistochemical assays using Image J. Scale bar: 100 µm. ** represents *p* < .01, *** represents *p* < .001.

To further study the chemosensitization effect of didymin inclusion complexes, DOX was injected intraperitoneally at 5 mg/kg with or without orally administrated didymin/HP-β-CD in mice bearing resistant MCF-7/ADR tumor (Figure 6(A)). All DOX therapy could inhibit tumor growth (Figure 6(B–D)). Compared with our previously reported results (Qing et al., 2019), DOX alone exert weak inhibitory effects on tumor growth, indicating that the MCF-7/ADR tumor model possesses the drug resistance toward the chemotherapeutics. Compare to the DOX group, the addition of didymin/HP-β-CD exhibited much stronger tumor growth inhibition effect (34% vs. 63%). With DOX treatment, the weight of both treated groups showed significant weight loss (Figure 6(E)). However, there was no significant body weight change observed between the DOX group and DOX + Didymin/HP-β-CD group. Furthermore, H&E staining of the major organs indicated that there is no abnormal pathology or noticeable organ damage induced by various formulations (Figure S5). These results jointly indicated that Didymin/HP-β-CD could effectively inhibit the chemoresistance development in tumors, thereby overcoming MDR. It is worth mentioning that this safe and effective oral administration strategy greatly promotes the feasibility of this supplementary treatment of didymin.

To further verify the synergistic effect of didymin/HP-β-CD with DOX on the growth of the resistant tumor, the tumor tissues were analyzed with H&E staining, immunostaining staining (Ki67) and TUNEL assay as shown in Figure 6(F). The H&E staining of tumor cells in the untreated mice showed classic structural features of tumor tissue, including disordered alignment, different cell sizes, and morphologies, large round to oval nuclei and high nuclear/cytoplasmic ratios. However, in the combination of DOX and didymin/HP-β-CD, significant tumor-killing activity was evident, including the appearance of a large area of necrotic tumor area, the nuclei became small and shrunken, and distinct apoptotic traits appeared. The expression of Ki-67 is highly associated with tumor cell proliferation and has been widely used as a marker of proliferating cells in the routine pathological exam (Xu et al., 2016). The brown spots showed minimal expression of Ki67 protein in the combination of DOX and didymin/HP-β-CD, showing an ability to inhibit tumor proliferation (Figure 6(F,G)). The TUNEL assay (Figure 6(F,H)) also produced consistent results with Ki67 results. The DOX and didymin/HP-β-CD groups showed the most significant inhibition of drug resistance in tumors (Figure 6(F–H)). These data indicate that Didymin/HP-β-CD could effectively overcome the resistance of tumor tissues to DOX, inhibit tumor proliferation, and increase the number of apoptotic cells after treatment.

## 4. Conclusion

In this study, we formulate the didymin by the cyclodextrin inclusion complex strategy to improve its solubility and bioavailability and develop an orally administrated adjuvant drug to combat MDR. The didymin inclusion complex was successfully prepared through a classic saturated aqueous solution method. Experimental results demonstrated that both HP-β-CD and β-CD were able to form the didymin inclusion complex, and increased the solubility and bioavailability. More importantly, the formation of didymin inclusion complex could also exert chemosensitive activity and enhance the DOX cytotoxicity. These obtained results indicated that the cyclodextrin complex could be an effective strategy to formulate didymin and offer a practical oral preparation for future adjuvant cancer therapy.

## Supplementary Material

Supplemental Material
