# Supplementary material for: In vitro and in vivo evaluation of didymin cyclodextrin inclusion complexes: characterization and chemosensitization activity
Source: Drug Deliv. 2019 Dec 20;27(1):54–65. doi: 10.1080/10717544.2019.1704941 (PMC6968488; doi:10.1080/10717544.2019.1704941)
Supplement: Supplemental Material [file IDRD_A_1704941_SM8274.docx]

**Supporting Information**

In vitro and in vivo evaluation of didymin cyclodextrin inclusion complexes: characterization and chemosensitization activity

1. **Materials and Methods**
   1. **Extraction and isolation**

The separation procedures and purity tests have been reported in our previous study. The separation procedure and purity test have been reported in our previous studies ([*1-3*](#_ENREF_1)). The air-dried and powdered plant material (15 kg) were extracted with 70% EtOH (2×100 L, each 2 h) at 80 C. Filtration and removal of EtOH from the extract under reduced pressure yielded a dark residue (3000 g), which was then suspended in H_2_O and extracted with petroleum ether, EtOAc, and n-butanol. The EtOAc extract (500 g) was chromatographed on a silica gel column (1.8 kg, 100–200 mesh, 20×70 cm) with gradient mixtures of CHCl_3_/MeOH (from 1:0 to 1:1, flow rate 60 mL/min). Eleven fractions were collected and examined by TLC on silica gel. Fraction D was purified with recrystallization with MeOH to isolate didymin (20 g). The purity test was performed with HPLC method. The batch number of didymin used in this study is 20180412 with 95.37% purity.

1.2 **Drug release study**

In vitro release study of didymin, didymin/HP-β-CD and didymin/β-CD were performed in a PBS dissolution medium at 37°C. Drugs were placed in a clean dialysis bag (with a cut-off molecular weight of 1000 Da), then suspended the dialysis bag in a centrifuge tube containing 40 mL PBS solution with stirring at a speed of 100 r /min. At a predetermined interval, take 1 mL of the PBS solution from the centrifuge tube for drug content measurement, and replenish an equal volume of fresh PBS solution into the beaker.

1. **Figures**

**
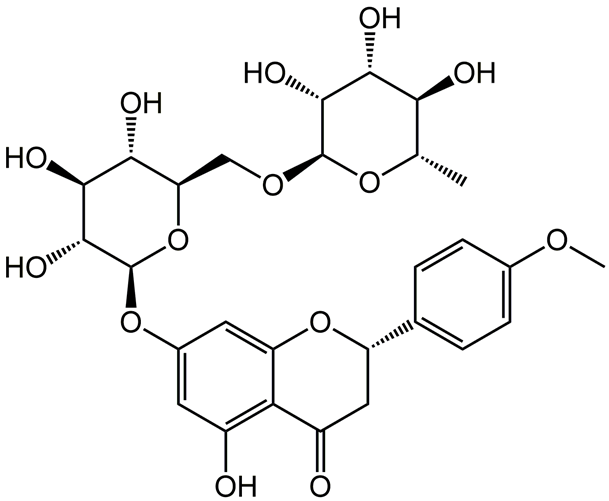
**

**Figure S1. The chemical structure of didymin**

**
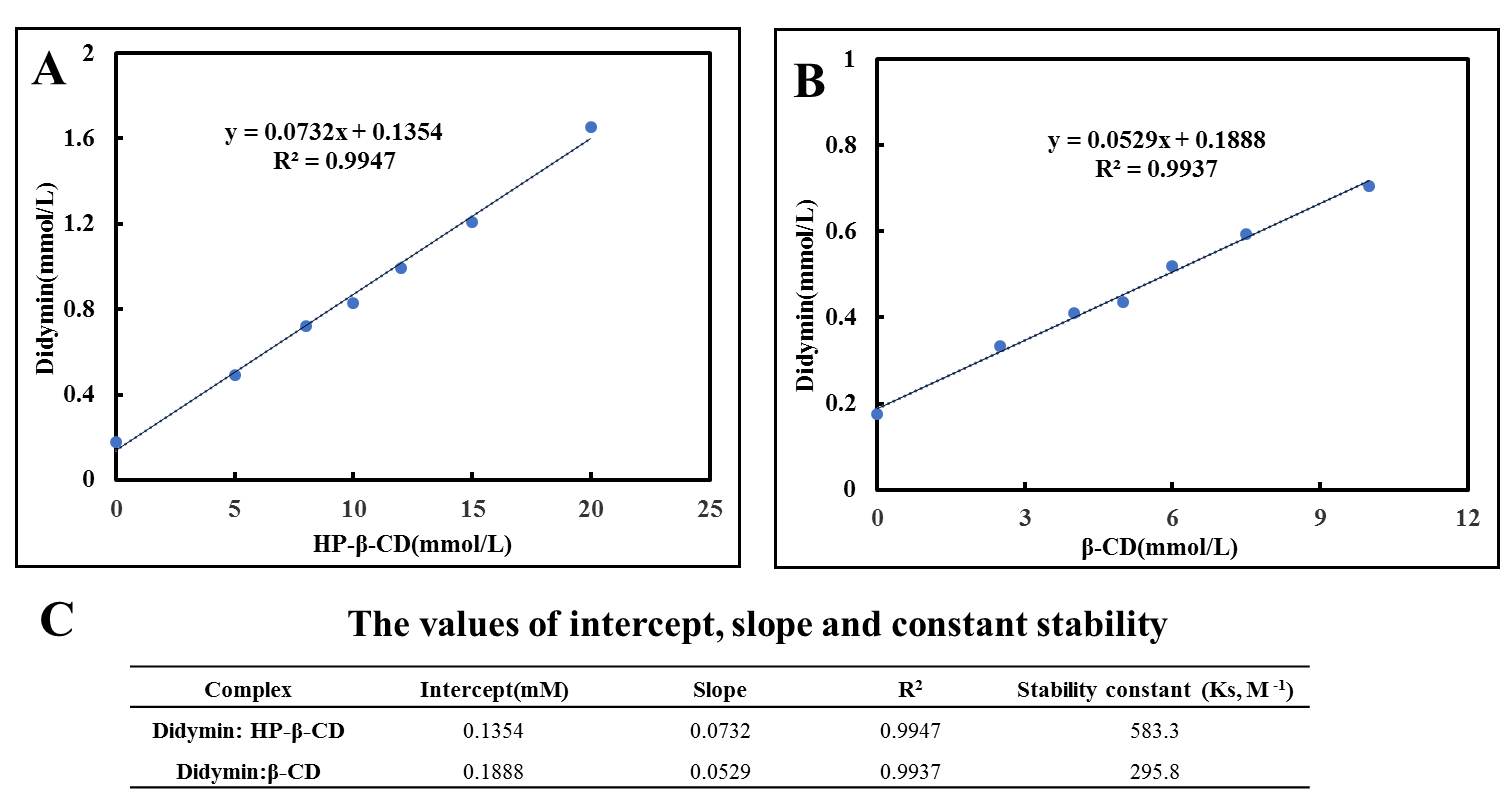
**

**Figure S2. Phase solubility studies of didymin with HP-β-CD (A) and β-CD (B) in distilled water and a summary of the calculated parameters (C).**

**
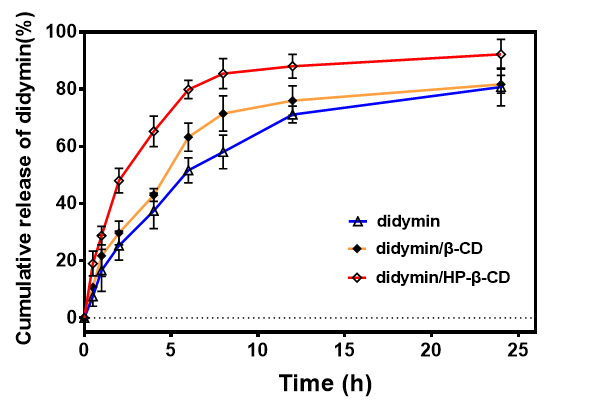
**

**Figure S3. The in vitro release behavior of didymin, didymin/HP-β-CD and didymin/β-CD in PBS. mean ± SD, n=3.**

**Figure S4. PXRD diffractogram of didymin/HP-β-CD, didymin/β-CD and stored for three months of didymin/HP-β-CD and didymin/β-CD.**

**
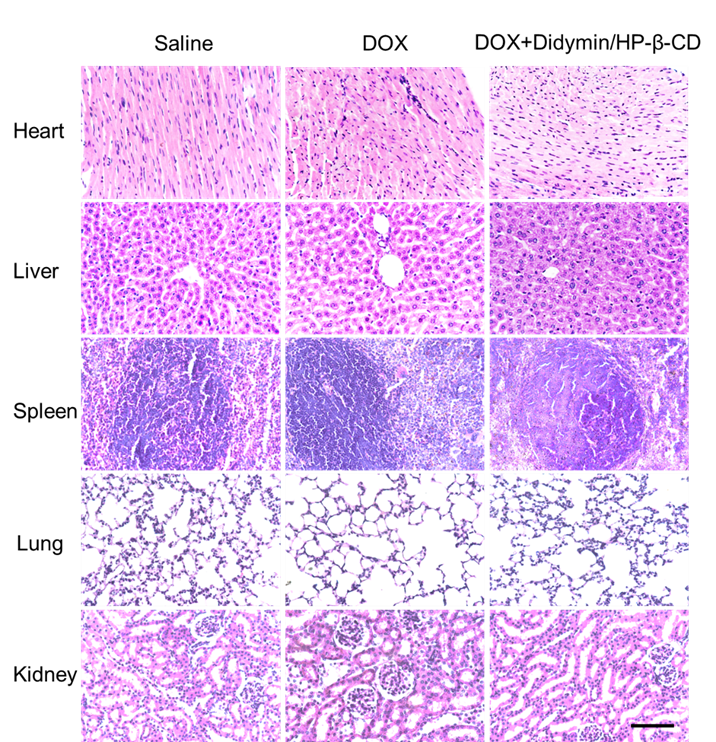
**

**Figure S5. H&E staining assay of major organs at the end of tumor growth inhibition studies. Scale bar: 100 µm.**

**Reference**

1. Wang, L. T., Sun, Z. H., Zhong, M. L., Wu, H. F., Zhang, H. J., Zhu, N. L., Sun, G. B., Ye, X. X., Xu, X. D., Zhu, Y. D., and Yang, J. S. (2017) Studies on chemical constituents of Clinopodium chinense, *Zhongguo Zhong yao za zhi = Zhongguo zhongyao zazhi = China journal of Chinese materia medica* *42*, 2510-2517.

2. Chen, R. C., Xu, X. D., Zhi Liu, X., Sun, G. B., Zhu, Y. D., Dong, X., Wang, J., Zhang, H. J., Zhang, Q., and Sun, X. B. (2015) Total Flavonoids from Clinopodium chinense (Benth.) O. Ktze Protect against Doxorubicin-Induced Cardiotoxicity In Vitro and In Vivo, *Evidence-based complementary and alternative medicine : eCAM* *2015*, 472565.

3. Zhu, Y. D., Hong, J. Y., Bao, F. D., Xing, N., Wang, L. T., Sun, Z. H., Luo, Y., Jiang, H., Xu, X. D., Zhu, N. L., Wu, H. F., Sun, G. B., and Yang, J. S. (2018) Triterpenoid saponins from Clinopodium chinense (Benth.) O. Kuntze and their biological activity, *Archives of pharmacal research* *41*, 1117-1130.
